# Neuroforensomics: metabolites as valuable biomarkers in cerebrospinal fluid of lethal traumatic brain injuries

**DOI:** 10.1038/s41598-024-64312-0

**Published:** 2024-06-13

**Authors:** Simone Bohnert, Christoph Reinert, Stefanie Trella, Andrea Cattaneo, Ulrich Preiß, Michael Bohnert, Johann Zwirner, Andreas Büttner, Werner Schmitz, Benjamin Ondruschka

**Affiliations:** 1 Institute of Forensic Medicine, University of Würzburg, Würzburg, Germany; 2 Department of Neurosurgery, University Hospital of Würzburg, Würzburg, Germany; 3 Institute of Legal Medicine, University Medical Center Hamburg-Eppendorf, Hamburg, Germany; 4 Department of Oral Sciences, University of Otago, Dunedin, New Zealand; 5 Institute of Forensic Medicine, Rostock University Medical Center, Rostock, Germany; 6 Institute of Biochemistry and Molecular Biology, Biozentrum, University of Würzburg, Würzburg, Germany

**Keywords:** Biomarker, Cerebrospinal fluid, Post mortem biochemistry, Forensic neuropathology, Traumatic brain injury, Metabolomics, Biochemistry, Biomarkers, Medical research

## Abstract

Traumatic brain injury (TBI) is a ubiquitous, common sequela of accidents with an annual prevalence of several million cases worldwide. In forensic pathology, structural proteins of the cellular compartments of the CNS in serum and cerebrospinal fluid (CSF) have been predominantly used so far as markers of an acute trauma reaction for the biochemical assessment of neuropathological changes after TBI. The analysis of endogenous metabolites offers an innovative approach that has not yet been considered widely in the assessment of causes and circumstances of death, for example after TBI. The present study, therefore, addresses the question whether the detection of metabolites by liquid-chromatography-mass spectrometry (LC/MS) analysis in post mortem CSF is suitable to identify TBI and to distinguish it from acute cardiovascular control fatalities (CVF). Metabolite analysis of 60 CSF samples collected during autopsies was performed using high resolution (HR)-LC/MS. Subsequent statistical and graphical evaluation as well as the calculation of a TBI/CVF quotient yielded promising results: numerous metabolites were identified that showed significant concentration differences in the post mortem CSF for lethal acute TBI (survival times up to 90 min) compared to CVF. For the first time, this forensic study provides an evaluation of a new generation of biomarkers for diagnosing TBI in the differentiation to other causes of death, here CVF, as surrogate markers for the post mortem assessment of complex neuropathological processes in the CNS (“neuroforensomics”).

## Introduction

Traumatic brain injury (TBI) is a common and often lethal result of accidents worldwide. It can occur in amateur and professional athletes, as well as during military exercises and combat missions and in the civilian sector after falls, acts of violence or car accidents among others^1–4^. Current estimates suggest that 69 million people worldwide suffer from TBI each year^5^. In the United States alone, the direct costs to the health system due to TBIs are estimated at 4 billion € per year^6^. The annual incidence of TBIs per 100,000 people is 939 cases worldwide, 1299 cases in North America, and 1012 cases in Europe^5^. In addition to research into the underlying pathological changes occurring in a TBI, both diagnosis and a reliable prognosis are highly relevant to provide people worldwide with optimized medical care in the clinical sector^7^.

After TBI, the blood-brain-barrier (BBB) and the cerebrospinal fluid (CSF)-brain-barrier (CSFB) can be impacted^8–12^. They become leaky, and, as a consequence, larger peptides, microbial metabolic substances, cytoskeletal and intracellular neuronal and glial elements can be identified in serum/plasma or in CSF samples by active and passive movements and be considered as biomarkers that act as surrogate markers of the central nervous system (CNS) neuropathology^6,13–19^. Metabolomics has altered the concept of a (single) ‘biomarker’. Traditionally, single biomarker analysis was the state of the art but the use of metabolic biopatterns (‘fingerprints’) has shown greater utility than a single biomarker in clinical application^19–24^.

Investigations of lethal TBI cases have always been a classical domain of forensic pathology with regard to traumatological and biomechanical aspects as well as in the case assessment^25^. Currently, autopsy and histological examination of the traumatized tissues are the most important investigations used in the post mortem routine to evaluate lethality and survival time (wound age)^26^. However, it can be challenging to recognize TBI at post mortem when macroscopic signs of head impacts such as contusions or lacerations are lacking^27^. Post mortem biochemical analyses are a promising objective resource for forensic pathologists to diagnose lethal TBIs. On that basis several forensic groups have already explored the potential to use TBI biomarkers for forensic purposes in a post mortem setting^27–30^.

Forensic medicine is a multidisciplinary branch of science which constantly strives to implement novel technologies from clinics into post mortem setting. State of the art “omics” technologies have begun to be performed in forensic medicine, particularly regarding the estimation of post mortem interval, diagnosis of intoxication and detection of drug abuse^31–34^. Since metabolomics is nearly untouched in the assessment of causes and circumstances of death, for example TBI, the analysis of endogenous metabolites offers an innovative investigative approach, which may become a diagnostic additive to the forensic methodological spectrum of neuropathological processes in the future (“neuroforensomics”)^13^.

For the first time, this forensic study will provide an evaluation of a new generation of biomarkers for diagnosing TBI and associated neuropathological changes in the CNS in the differentiation to another cause of death, namely acute cardiovascular control fatalities (CVF) and to check for significant differences in metabolites.

## Results

### Significantly changed metabolites

**Figure 1:**
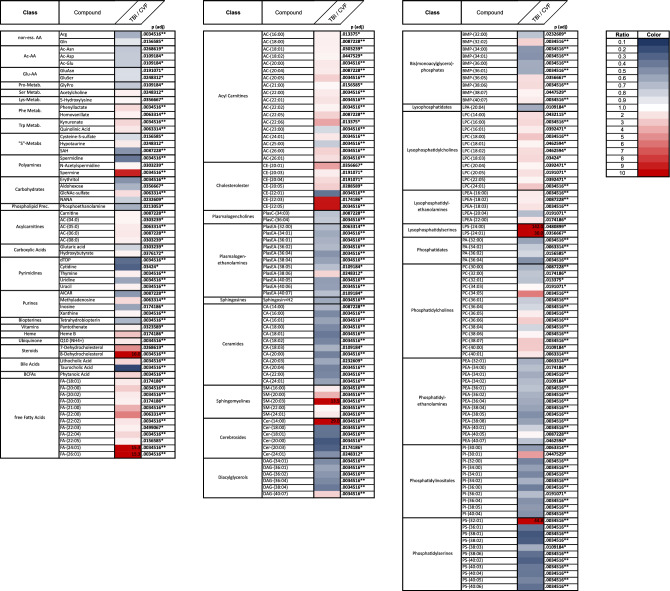
Heatmap of all 212 significantly changed metabolites between traumatic brain injury (TBI) and cardiovascular failure (CVF) as cause of death. In order to compare the metabolite distribution within the two cohorts studied, the quotients of the metabolite concentrations (i.e., metabolite A in the CSF of persons who died of TBI to the concentration of metabolite A in the CSF of persons who died of CVF) were presented in the form of a heat map. All values between 0.10 and 10.00 were assigned different color gradations. Values outside this defined range were shown with the numerical quotient values in addition to the color coding.

In the present study, 212 metabolites were identified in post mortem CSF that showed significant concentration differences in lethal TBI compared with the control group (CVF) irrespective of a potential PMI influence (Fig. 1), for cohort details see Table 1. Exemplarily, the metabolites 8-Dehydrocholesterol, FA-(24:01), FA-(26:01), SM-(20:03), Cer-(14:00), LPS-(24:00), LPS-(24:01) and PS-(32:01) were detected in heavily elevated (as minimum tenfold higher) CSF concentrations compared with CVF (Fig. 1). In contrast, some metabolites also showed intensively decreased TBI concentrations compared to CVF such as cytidine, taurocholic acid, Cer-(20:00), Cer-(20:03), PS-(38:01), PS-(38:02) (Fig. 1). Supplementary Fig. 1 shows a heatmap of all tested metabolites with regard to differences between TBI and CVF as well as correlation of metabolites level to PMI.

**Table 1:** Cohort summary of analyzed cases.

	Cases			Age [years]			PMI [hours]			Brain weight [gram]			Heart weight [gram]			CPR		Mechansim of death		
	Total	Male	Female	Mean	SD	Range	Mean	SD	Range	Mean	SD	Range	Mean	SD	Range	Yes	No	traffic accident	fall with blunt trauma	sudden cardiac death
TBI	30	21	9	67	21	19–92	125	48	32–199	1338	182	1060–1655	470	129	250–660	12	18	7	23	0
CVF	30	20	10	68	17	33–93	110	61	35–309	1392	150	1000–1670	541	108	360–855	19	11	0	0	30
Mann–Whitney-U				*p* = 0.8978			*p* = 0.1331			*p* = 0.2118			*p* = 0.0574							

### Formation of a TBI detecting quotient

In search of predictive metabolites in post mortem CSF, the combination of the metabolites “SM-(20:03)”, “LPS-(24:00)”, “LPS-(24:01)”, “PS-(32:01)” and “8-dehydrocholesterol” in the numerator and “Taurocholic Acid”, “Cer-(20:00)”, “Cer-(20:03)”, “PS-(38:01)” and “PS-(38:02)” in the denominator proved best to diagnose the presence of a TBI. The combination of the quotient is based on the five highest and five lowest ratios between TBI and CVF and includes statistically significant parameters only. The quotients of the TBI cases formed a mean value of 729.659 (minimum value: 0.719; maximum value: 9567.149), while the quotients of the CVF cases showed a mean value of 1.484 (minimum value: 0.069; maximum value: 8.584).

**Figure 2:**
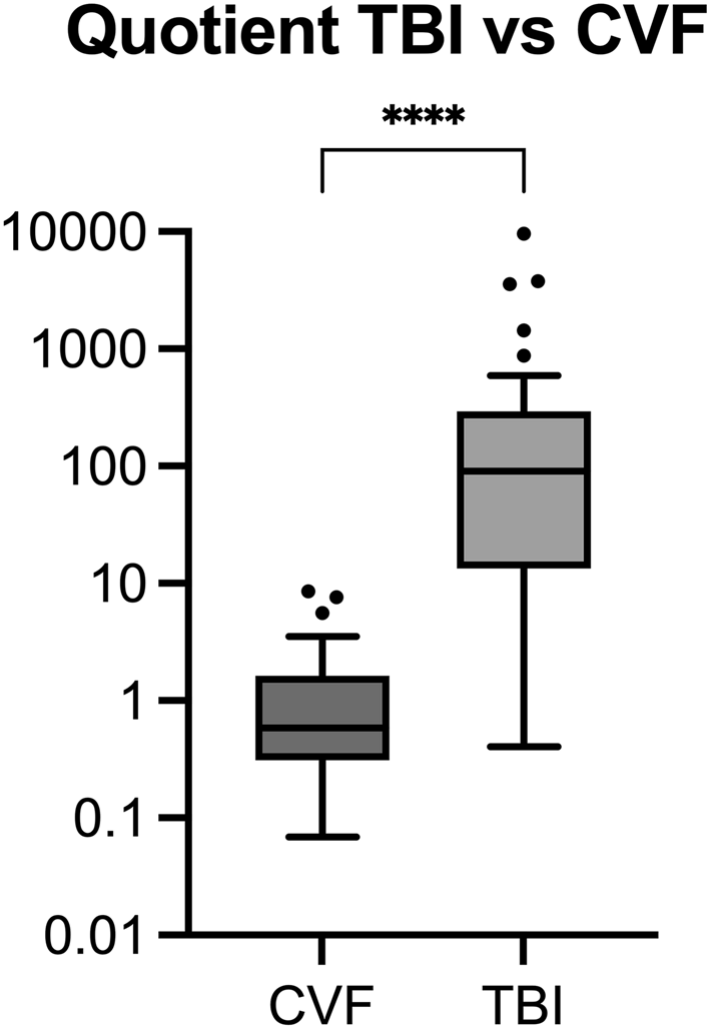
Boxplot calculated quotient values grouped according to cause of death. Mann–Whitney U test: ****p < 0.001. The boxes indicate the 25th percentile, median and 75th percentile. Whiskers depict the minima and maxima excluding outliers (Tukey style).

A cut-off value of ≥ 8.626 was chosen for identifying the presence of a TBI by using receiver operating characteristic curve and Youden index. 93.3% of the TBI cases examined were correctly diagnosed using this cut-off vale (93.3% sensitivity, 100% specificity). The CVF samples 32 and 33 were assigned to the CVF cases as a false negative (false negative rate: 6.7%). This showed that with 100% sensitivity the control group was correctly categorized as CVF (see Fig. 2).

### Formation of a control quotient

**Figure 3:**
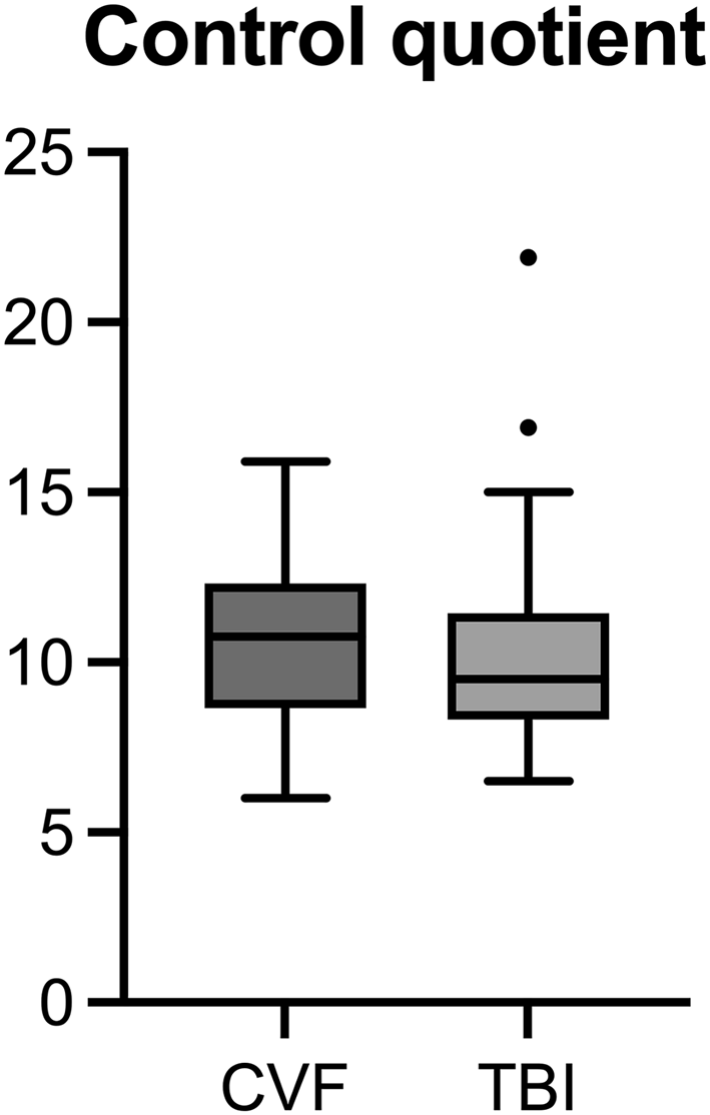
Boxplot and column chart of calculated control quotient values grouped according to cause of death. Mann–Whitney U test: *p* > 0.05. The boxes indicate the 25th percentile, median and 75th percentile. Whiskers depict the minima and maxima excluding outliers (Tukey style).

Metabolites—here eight non-essential amino acids in alphabetical order as potential alternative—were randomly selected to form a metabolite combination control group. The combination of metabolites was composed of “alanine, arginine, asparagine, aspartic acid” in the numerator and “cysteine, glutamic acid, glutamine, glycine” in the denominator. Here, the quotient of TBI CSF samples as autopsy-confirmed cause of death formed a mean value of 10.3 (minimum value: 6.495; maximum value: 21.936), while the quotient of the CVF cases formed a mean value of 10.6 (minimum value: 6.009, maximum value: 15.891). This resulted in 28 overlaps of the quotient value, which thus did not allow any valid conclusions to assign the cause of death by CSF metabolite profiles (see Fig. 3).

Further comparison of both quotients was made between the quotients of the TBI cases and the quotients of the CVF cases, and between the “control-quotients” of both cohorts. There was a highly significant difference between the quotient of the TBI and CVF group (*p* < 0.001). In contrast, the non-essential amino acids failed to distinguish between the two different causes of death (*p* > 0.05).

During sample preparation, increased red staining of the TBI CSF samples was observed compared to CVF even in cases passing the visual examination during sample selection. Therefore, the detected peak areas of “heme B” from the final, factor-corrected data set were plotted together with the quotient to exclude a sole influence of hemolysis metabolites on the quotient. The peak area of “heme B” did not correlate with the level of the quotient, so that no conclusion regarding a TBI could be drawn by merely inspecting the CSF sample visually.

## Discussion

In the present study, numerous metabolites were identified in post mortem CSF that showed significant concentration differences in lethal TBI samples compared with CVF and may become a diagnostic additive to the forensic methodological spectrum of neuropathological processes (“neuroforensomics”).

### Metabolites in cardiovascular fatalities

To our knowledge, no scientific studies are currently available on the correlation of post mortem CSF metabolites in CVF, so only general findings on metabolites in blood—and not in CSF—can be used here as reference values. In this context, the large number of altered metabolites from the group of lipids appeared particularly interesting. Long-chain free fatty acids can penetrate the intact cell membrane of cardiomyocytes by passive diffusion^35–37^ or by means of active transport^38–40^ and thus reach the cytoplasm. Downregulation of fatty acid oxidation in the heart and an increasing number of free fatty acids in the blood have been reported in CVF^41–45^. In contrast, an increased uptake of fatty acids from the blood into the cells has also been observed in the preliminary stages of heart failure, e.g., cardiac insufficiency^46,47^. Because of these ambiguities and inconsistencies, the interpretation of free long-chain fatty acid measurements in heart failure is difficult and should be the subject of further research. Moreover, an increase in plasma spermidine and phosphatidylcholine concentrations has been described in heart failure^48^, which has also been demonstrated in the CSF of the CVF group in the given study. In general, many metabolomic studies can be found in association with heart failure. However, varying metabolites are frequently described in elevated or decreased concentrations^48–51^, which may be explained both by heterogeneity of the test material and by different pathomechanisms of the disease and, when investigating post mortem samples, various agony periods.

### Lipophilic metabolites in traumatic brain injury

In the present study, some classes of metabolites, such as lysophospholipids (LP), LPC, PS, PC, PEA, and SM, showed remarkable concentration differences even within one grouped cause of death. Cholesterol, sphingolipids and phospholipids are always present in the human CNS^52–54^. After fatty tissue, the brain shows the highest concentrations of various lipids in the body^55^, which is why a TBI leads to an increased release of these substances^56^. Consequently, brain tissue also contains more lipids than the heart, which is why their concentrations in the CSF are to be expected as increased in TBI compared with CVF. This assumption is confirmed in the present paper by a multitude of altered lipids. Thus, the increases in the measured values of long-chain free fatty acids (both FA-(24:01), FA-(22:00)) and sphingolipids (here SM-(20:03)) described in the literature after TBIs could also be detected and confirmed in this study^55^. Interestingly, different fatty acids are present in the different regions of the brain in different configurations and with divergent side chains^53–55^. However, the current research situation does not allow any conclusions to be drawn regarding the assignment of certain lipids to defined brain regions, which is why it was impossible to draw any conclusions as to the origin of the metabolites in this paper. Nevertheless, one may possibly assume that the increased detection of partially long-chain, partially short-chain fatty acids in the present study occurs in dependence on their temporal entry into the CSF. Pasvogel et al.^57^ and Sarkar et al.^58^ described a maximum LPC level one day after trauma in lethal TBIs clinically and a general increase in the substance class of LPs after a TBI. In contrast, maximum concentrations of PS, PC, PEA, and SM in the CSF were not measured before the fourth posttraumatic day^57^. Bearing in mind the maximum survival time of 90 min in the here investigated cohorts, some metabolite changes definitively seem to occur beyond this time frame and must be investigated in future studies.

### Metabolites of tryptophane metabolism

In the group of lethal TBIs, kynurenic acid and other metabolites of the tryptophane metabolism (kynurenine) were found to be significantly increased. It was demonstrated before that elevated levels of kynurenine, in addition to IL-6 or TNFα, indicate an inflammatory event in the brain^59,60^.

### Spermidine

Reports of decreased spermidine levels after TBI^61^ in combination with the aforementioned increased concentration in cardiac insufficiency can also be confirmed with the given results. Spermidine is considered an important metabolite for successful cell growth and is involved in DNA replication, protein synthesis and lipid stabilization^62,63^. Huang et al.^61^ assumed that spermidine substitution may positively influence the course of a TBI and lead to an increased regenerative capacity of the human organism. In addition, these authors describe a relationship between the severity of a TBI and a significant decrease in spermidine levels, which means that the metabolite can be used as an indicator of the extent of a TBI. Apart from decreased spermidine levels, increased spermine levels were detected in CSF after TBI in the present study.

### Glutamylcysteine and cysteine

In the present study, glutamine and glutamate, both precursors of glutathione, were present in lower concentrations after TBI compared with CVF. Glutathione is an important radical scavenger of the body, which binds reactive oxygen molecules by oxidation. It thus counteracts oxidative stress after a TBI and protects cellular molecules from harmful radicals^64,65^. The metabolites can also protect human cells from reactive oxygen molecules^66^. Reported decreases in glutathione concentration in the CSF after TBI^67^ can also be attributed in this study to corresponding likewise decreased values measured for glutathione precursors.

### Sphingomyelin and ceramide

Ceramides play an important role as signaling molecules in cell stress, apoptosis, cell cycle arrest and in processes of cell differentiation^68,69^. Furthermore, ceramides can be converted into SMs or also sphingosines^70^. The low concentrations of ceramides measured after a TBI, in combination with increased SM concentrations, could indicate that a conversion of ceramides into SMs has taken place. Thus, an increase in SM concentrations in the CSF, whether by de novo synthesis or conversion of ceramides, could also be considered to represent a physiological regeneration effort of the human organism. Consequently, decreased ceramide values in combination with increased SMs could be the result of regenerative metabolic processes, which might take place after a TBI.

### Lysophosphatidylcholine

In the TBI group, many elevated LPCs were found compared with the control group. Increased concentrations of LPCs after TBI have already been reported in clinical setting^58,71^. One theory to explain the increased values describes a bidirectional feedback system between the brain and the liver. In the context of a TBI, this leads both to increased enzymatic formation of LPC from PC in the CNS and also to increased release of LPC by the liver^72–74^. Both mechanisms in this “brain-liver axis” cause an increase of the metabolite after a TBI, which in turn induces cognitive dysfunctions and inflammatory processes in the brain^71,75,76^.

### Erythritol

In the present data set, erythritol, which chemically belongs to the sugar alcohol group, was detected in reduced concentrations in the CSF after TBI compared to the CVF control group. One possible explanation for the decreased concentration of erythritol after TBI is that it reflects a reactive response of the human organism to increased intracranial pressure^77^.

### Metabolites of the cholesterol metabolism

The most significant increase among the TBI samples was detected in 8-dehydrocholesterol, a reactant in cholesterol synthesis. This observation supports the assumption that cholesterol metabolism and pathological changes after traumatization are closely linked^78^.

### Interpretation of the quotient-values

When calculating the TBI-CVF quotient, sample number 14 of the CVFs turned out to be elevated and consequently would be assigned to a potential TBI case as a false result. However, a more detailed analysis of the circumstances of the subject's death revealed a special case constellation that could potentially account for the increased quotient. According to the police report and the statements of witnesses, the deceased had suddenly fallen from his bicycle and died despite resuscitation measures. Autopsy found a CVF in the presence of a severely pre-existing heart disease; nevertheless, the values measured in the CSF indicate that superficial skin abrasions of the head may be considered as residues of shearing movements of the head as head concussion, which may have promoted an increase of metabolites in the CSF before death. This special case constellation thus shows a weakness of the quotient calculated in this study, which requires a critical assessment of the exact circumstances of death in the context of an autopsy. CSF measurement therefore adds in diagnosing TBI but should not be interpreted blinded to the case details.

## Limitations

Despite the matching of the individual’s variables listed in Table 1, no distinct homogeneity could be established between both groups TBI versus CVF. The most significant influencing aspect in post mortem studies is the PMI. In order to minimize the influence of this distorting factor, the PMI was matched in the present study between cohorts and correlated to all single metabolites.

The validity of the results can only be assessed to a limited extent, as the study included a total of 60 samples only mainly due to economic restrictions and compared TBI to only one group of natural circumstances of cardiac death, ignoring other common death mechanisms (inflammatory, malignancy, exsanguination, non-TBI trauma and others). The same applies to TBI where blunt force trauma cases were selected. To keep the study within an economically scope the definition of only one trauma group, TBI, was necessary. It would be important and interesting to benchmark our findings to another trauma situation e.g. orthopedic or isolated torso trauma in further studies. Moreover, a single center trial with 30 TBI versus 30 CVF individuals cannot be considered robust enough to establish a quotient for detecting TBI related deaths with certainty. The usefulness of the method must be proved in following studies with sufficient sample sizes.

Another point of criticism is the unblinded sample evaluation, which is why an investigator bias cannot be ruled out. Further potential influencing factors of the metabolite concentrations such as body mass index, high blood pressure, kidney functions, medication taken or other previous diseases during the lifetime of the deceased could not be traced back from the files of the Institute of Legal Medicine of the University of Würzburg. Therefore, there may also be distortions of the measurement results here. In addition, the interpretation of the measured metabolite concentrations is hampered by the limited availability of study results in humans yet, which is especially true for the post mortem field. Some of the physiological responses to a TBI have only been described using mouse models so far and are therefore of limited relevance to the human body. As until now, metabolomics has hardly been applied in legal medicine^79^, the present study lacks established cut-off values and reference ranges, but the results presented here could be a starting point for this. To avoid any incongruencies, the single cut-off value was only used descriptively and must be checked for practical applicability in further studies.

The measurement method itself is not suitable for the absolute quantification of metabolites without the addition of stable isotope-labeled standard substances. To avoid this problem, the signal areas obtained for each metabolite were expressed as a percentage of the sum of the areas of all verified signals (i.e. normalized to the total metabolites). For this reason, no detection limits can be specified for the individual metabolites. These are not only different for each metabolite, but also depend on the composition of the respective samples.

## Conclusion

This study focused on a new generation of potential biomarkers in CSF samples, endogenous metabolites, which offer an innovative and promising investigative approach (“neuroforensomics”). Based on the emerged results that these metabolites showed validity for diagnosis causes of death such as TBI versus CVF, they confirm the important role of thanatobiochemistry and analytical techniques applicable in forensic pathology, which is why further associated studies are crucial.

## Material/methods

### Sample collection, assessment and selection

The sample set investigated included 30 cases of CVFs as a specific example of sudden death due to an internal cause (10 females and 20 males aged between 19 and 93 years) and 30 cases of acute death after TBI in sex- and age-matched collection (9 females and 21 males aged between 19 and 92 years), all autopsied at the Institute of Forensic Medicine of the University of Würzburg. The brain weight of all deceased was between 1000 and 1670 g (mean 1365 g, SD 169). In the group of lethal TBI, it was between 1060 and 1655 g (mean 1338 g, SD 150); in the cohort of CVF, it ranged between 1000 and 1670 g (mean 1392 g, SD 182). All included CVF cases died suddenly or were found dead after only a short unobserved period of time. The survival time of TBI cases ranged between ‘none’ (immediately dead) in cases of open TBI / pons dehiscence up to 1.5 h in cases of cortical contusions with final but unsuccessful cardiopulmonary resuscitation efforts in 12 of 30 TBI cases. The post mortem interval at autopsy of all cases ranged between 32 to 309 h (mean 118 h, SD 56). More detailed characteristics are displayed in Table 1 as cohort summary and in Supplementary Table 1 in case-per-case tables.

This study has been approved by the ethics committee of the Medical Faculty of the University of Würzburg (local number 203/15). Following the approval of the ethical committee, the next of kin of the deceased (when personally known) or the public prosecutor’s office Würzburg (if no relatives were available) gave their informed consent to analyze the CSF samples, which were anonymized according to the guidelines from the central ethic commission of the federal medical association. All methods were carried out in accordance with the relevant guidelines and regulations.

### Preparation

Collection of CSF by suboccipital puncture using a disposable needle during dissection of the head and storage the body fluid aspirated in polypropylene test vessels. Cases with obviously bloody / reddish CSF were excluded after an indicative quote of the samples for their optical appearance. Centrifugation of the sample was performed directly after collection at 5000 rpm for 5 min at 4 °C. Until further laboratory processing, the CSF supernatant was stored at − 80 °C.

### LC/MS analysis of water-soluble metabolites

**Figure 4:**
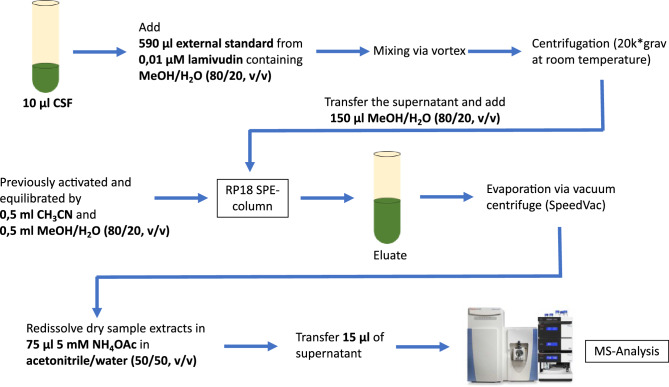
Illustration of the extraction steps of water-soluble metabolites from CSF in preparation for mass spectrometry analysis using MeOH/H_2_O extraction. Use of the Thermo Scientific Q Exactive HF-X-image with kind permission of Thermo Fisher Scientific^80^.

The analysis of the water-soluble metabolites is based on MeOH/H_2_O extraction and pre-purification by solid phase extraction with subsequent analysis by LC/MS. 10 µl of the sample to be tested and 590 µl of an external standard, in this case 0.01 µM lamivudine in MeOH/H_2_O (80/20, v/v), were mixed. An RP18 SPE column was first activated by elution of 0.5 ml CH_3_CN and then equilibrated by 0.5 ml MeOH/H_2_O (80/20, v/v). The sample was then centrifuged (20 k × grav at room temperature) and the supernatant was transferred to the equilibrated RP18 SPE column. The resulting eluate was collected in an Eppendorf reaction vessel. For post-elution, another 150 µl MeOH/H2O (80/20, v/v) were added to the column. In the next step, the total eluate of the RP18 SPE column was evaporated by vacuum centrifugation (SpeedVac; Thermo Fisher Scientific, Waltham, USA). The residue was dissolved in 75 µl of 5 mM NH_4_OAc in acetonitrile/water (50/50, v/v) and 15 µl of the supernatant was transferred to a sample tube. The extraction of the water-soluble metabolites is graphically shown in Fig. 4.

Then, solvent A consists of 5 mM NH_4_OAc in acetonitrile/water (40/60, v/v), solvent B of 5 mM NH_4_OAc in acetonitrile/water (95/5, v/v). After loading 3 µl of sample onto an HILIC column (BEH-amide) at 30 °C, elution was performed by the following gradient: 100% solvent B for 2 min, followed by a linear decrease to 10% solvent B within 23 min, then maintaining 10% B for 16 min, then returning to 100% B in 2 min. The column was equilibrated with 100% solvent B for 7 min. The column temperature was set to 30 °C, the flow rate was maintained at 200 µL/min. The eluate was directed to the mass spectrometer (Q-Exactive Mass Spectrometer (QE-MS)) for analysis between 1.85 and 20.0 min.

### LC/MS analysis of lipophilic metabolites

**Figure 5:**
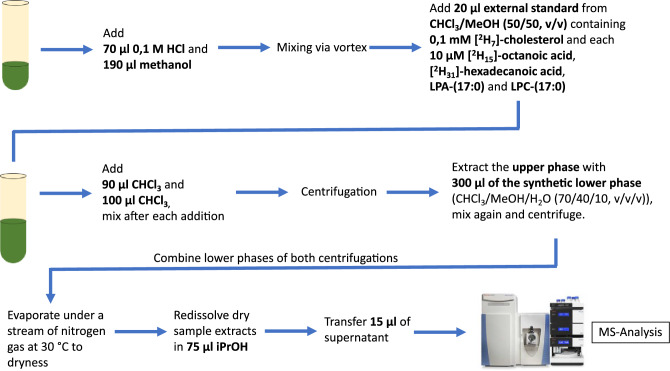
Illustration of the extraction steps of lipophilic metabolites from CSF in preparation for mass spectrometry analysis using a modified Bligh/Dyer (B/D) extraction. Use of the Thermo Scientific Q Exactive HF-X-image with kind permission of Thermo Fisher Scientific^80^.

The analysis of lipophilic metabolites is based on a modified Bligh/Dyer (B/D) extraction^81^. To 100 µl of the CSF to be examined, 70 µl of 0.1 M HCl and 190 µl of methanol were added in succession. After mixing the sample by homogenization, 20 µl of an external standard consisting of CHCl_3_/MeOH (50/50, v/v) in 0.1 mM [^2^H_7_] cholesterol and 10 µM each of [^2^H_15_]-octanoic acid, [^2^H_31_]-hexadecanoic acid, LPA-(17:0) and LPC-(17:0) were added. Subsequently, 90 µl of CHCl_3_ was added to the sample and mixed again by homogenization. After addition of another 100 µl CHCl_3_, the CSF sample was mixed again and then centrifuged. The resulting upper phase was transferred to a new Eppendorf reaction vessel and extracted again with 300 µl synthetic subphase (CHCl_3_/MeOH/H_2_O (70/40/10, v/v/v)). The resulting solution was mixed by homogenization and then centrifuged. The upper phase was discarded, the lower phases from the two centrifugations were combined, centrifuged and finally transferred to a new Eppendorf reaction vessel. The sample was then concentrated under a constant flow of nitrogen at 30 °C for drying. For LC/MS analysis, the residue was dissolved in 75 µl iPrOH and 15 µl of the supernatant was transferred to a sample vial. The extraction of the lipophilic metabolites is graphically shown in Fig. 5.

The parameters and procedures of the LC/MS used were solvent A consisting of MeOH/H_2_O/FA (5/94.9/0.1, v/v/v), and solvent B of CH_3_CN/iPrOH/H_2_O/FA (45/45/9.9/0.1, v/v/v/v). After loading 3 µl of sample onto a C8 column at 40 °C, elution was performed by the following gradient: 20% solvent B for 2 min, followed by a linear increase to 100% solvent B within 5 min, then maintaining 100% B for 33 min, then returning to 20% B in 1 min. Equilibration of the column was performed with 20% solvent B for 5 min. The column temperature was set to 40° C, the flow rate was maintained at 350 µL/min. The eluate was directed to the QE-MS for analysis between 2.0 and 38.0 min.

### MS parameter settings

The following MS scan settings were used for the analysis of the water-soluble metabolites: Scan type: Full-MS in pos./neg. mode; Scan range: 69.0–1000 m/z; Resolution: 70 k; AGC target: 3E6; Maximum injection time: 200 ms. For the analysis of lipophilic metabolites, the following MS scan settings were applied: Scan type: Full-MS in pos./neg. mode; Scan range: 200–1650 m/z; Resolution: 70 k; AGC target: 3E6; Maximum injection time: 200 ms.

### Ion source parameters

The ion source parameters for analysis of the water-soluble metabolites were defined as follows: Sheath gas flow rate: 20; Auxiliary gas flow rate: 1; Sweep gas flow rate: 0; Spray voltage: 3.0 kV; Capillary temperature: 320 °C; S-lens RF level: 50.0; Aux gas heating temperature: 120 °C. For lipophilic testing, the following changes must be made to the Heated Electrospray Ionization (HESI) settings: Sheath gas flow rate: 30; Auxiliary gas flow rate: 10; Sweep gas flow rate: 0; Spray voltage: 3.0 kV; Capillary temperature: 320 °C; S-lens RF level: 55.0; Aux gas heating temperature: 120 °C.

### Data analysis and processing

Annotation and quantification of the obtained signals based on the calculated monoisotopic metabolite masses (MIM +/− H+  ± 3 mMU) was performed using TraceFinder™ software version 3.3 (Thermo Fisher Scientific, Waltham, USA).

### Materials

LC/MS analysis was performed using the Thermo Scientific Dionex Ultimate 3000 UHPLC system coupled to a QE-MS with a HESI probe (Thermo Fisher Scientific, Waltham, USA). A Javelin filter for 2.1 mm (Thermo Fisher Scientific) was used as a particle filter. HILIC-column: XBridge Premier BEH Amide (2.5 µm, 2.1 × 100 mm) (Waters, Milford, USA). C8-columns: Acclaim 120 C8 precolumn (5 μm particles, 10 × 2 mm) combined with an Acclaim RSLC 120 C8 (2.2 μm particles, 50 × 2.1 mm) (Thermo Fisher Scientific). The ultrapure water came from a Millipore water purification system (Milli-Q Merck Millipore, Darmstadt, Germany). HPLC-MS solvent, LC/MS-NH_4_OAc, chemicals, standard and reference compounds were available commercially (Merck Millipore).

### Evaluation of the MS dataset and statistical analyses

To compare the metabolite composition of the 60 CSF samples studied, the percentage of the signal area of a particular metabolite (water-soluble or lipophilic) was first calculated from *all* water-soluble or lipophilic metabolites measured for each sample. The resulting values were then used to calculate the mean value and standard deviation of samples from CVF and TBI groups, respectively.

In the next step, each “TBI mean” of a metabolite was divided by the corresponding “CVF mean” and thus a quotient was formed that depicts the ratio of a metabolite between lethal TBI and acute CVF death (see heatmap for details), where a ratio > 1 means a higher content of the metabolite in question in TBI compared to CVF cases and vice versa. However, in the absence of comparative literature and a scientific gold standard, the range of values was set arbitrarily.

Data analysis and visualization was conducted using Microsoft Excel version 16.15 (Microsoft Corporation, Redmond, USA), IBM SPSS Statistics V27.0 (IBM Corp., Armonk, USA), and Prism version 9 (GraphPad Software Inc., La Jolla, USA). After checking the data for normality using significant Shapiro-Wilk tests, we ran non-linear evaluations with Mann-Whitney U tests for calculations between both groups TBI and CVF. Further, a Benjamini-Hochberg procedure was calculated to avoid type I error accumulation. Adjusted *p* values of 0.05 or less were considered as statistically significant. Correlations between metabolites levels and PMI were computed using Spearman’s correlation coefficients using the same significance level. ROC curve analysis was used to identify the sensitivity and specificity of threshold values for the differentiation between TBI and CVF by conservative estimations.

### Supplementary Information

Supplementary Information.

## Data Availability

The raw datasets used and analysed during this study are available from the corresponding author on reasonable request.

## References

[1] Ling H, Hardy J, Zetterberg H (2015). Neurological consequences of traumatic brain injuries in sports. Mol. Cell. Neurosci..

[2] Menon DK, Maas AI (2015). Traumatic brain injury in, 2014. Progress, failures and new approaches for tbi research. Nat. Rev. Neurol..

[3] Nakagawa A (2011). Mechanisms of primary blast-induced traumatic brain injury: Insights from shock-wave research. J. Neurotrauma.

[4] Risdall JE, Menon DK (2011). Traumatic brain injury. Philos. Trans. R. Soc. Lond. B Biol. Sci..

[5] Dewan MC (2018). Estimating the global incidence of traumatic brain injury. J. Neurosurg..

[6] Wang KK (2018). An update on diagnostic and prognostic biomarkers for traumatic brain injury. Expert. Rev. Mol. Diagn..

[7] Maas AIR (2017). Traumatic brain injury: Integrated approaches to improve prevention, clinical care, and research. Lancet Neurol..

[8] Ballabh P, Braun A, Nedergaard M (2004). The blood-brain barrier: An overview: Structure, regulation, and clinical implications. Neurobiol. Dis..

[9] Tarasoff-Conway JM (2015). Clearance systems in the brain-implications for Alzheimer disease. Nat. Rev. Neurol..

[10] Agoston DV, Shutes-David A, Peskind ER (2017). Biofluid biomarkers of traumatic brain injury. Brain Inj..

[11] Daneman R, Prat A (2015). The blood-brain barrier. Cold Spring Harb Perspect. Biol..

[12] Ren Z (2013). ‘Hit & run’ model of closed-skull traumatic brain injury (tbi) reveals complex patterns of post-traumatic aqp4 dysregulation. J. Cereb. Blood Flow Metab..

[13] Bohnert S (2021). Metabolomics in postmortem cerebrospinal fluid diagnostics: A state-of-the-art method to interpret central nervous system-related pathological processes. Int. J. Legal Med..

[14] Katayama Y, Becker DP, Tamura T, Hovda DA (1990). Massive increases in extracellular potassium and the indiscriminate release of glutamate following concussive brain injury. J. Neurosurg..

[15] Osteen CL, Moore AH, Prins ML, Hovda DA (2001). Age-dependency of 45calcium accumulation following lateral fluid percussion: Acute and delayed patterns. J. Neurotrauma.

[16] Werner C, Engelhard K (2007). Pathophysiology of traumatic brain injury. Br. J. Anaesth..

[17] Zwirner J (2022). Forensic biomarkers of lethal traumatic brain injury. Int. J. Legal Med..

[18] Sieber M, Dreßler J, Franke H, Pohlers D, Ondruschka B (2018). Post-mortem biochemistry of nse and s100b: A supplemental tool for detecting a lethal traumatic brain injury?. J. Forensic Leg. Med..

[19] Zwirner J (2021). Assessing protein biomarkers to detect lethal acute traumatic brain injuries in cerebrospinal fluid. Biomolecules.

[20] Berger RP (2005). Serum neuron-specific enolase, s100b, and myelin basic protein concentrations after inflicted and noninflicted traumatic brain injury in children. J. Neurosurg..

[21] Berger RP (2006). Identification of inflicted traumatic brain injury in well-appearing infants using serum and cerebrospinal markers: A possible screening tool. Pediatrics.

[22] Papa L (2016). Time course and diagnostic accuracy of glial and neuronal blood biomarkers gfap and uch-l1 in a large cohort of trauma patients with and without mild traumatic brain injury. JAMA Neurol..

[23] Welch RD (2016). Ability of serum glial fibrillary acidic protein, ubiquitin c-terminal hydrolase-l1, and s100b to differentiate normal and abnormal head computed tomography findings in patients with suspected mild or moderate traumatic brain injury. J. Neurotrauma.

[24] Mondello S (2011). Blood-based diagnostics of traumatic brain injuries. Expert Rev. Mol. Diagn..

[25] Bratzke H, Püschel K (2011). Medikolegale begutachtung des schädel-hirn-traumas. Rechtsmedizin.

[26] Dettmeyer RB (2018). Forensic Histopathology. Fundamentals and Perspectives.

[27] Li DR (2006). Postmortem serum protein s100b levels with regard to the cause of death involving brain damage in medicolegal autopsy cases. Leg. Med. (Tokyo).

[28] Ondruschka B, Sieber M, Kirsten H, Franke H, Dreßler J (2018). Measurement of cerebral biomarkers proving traumatic brain injuries in post-mortem body fluids. J. Neurotrauma.

[29] Zwirner J (2021). Screening for fatal traumatic brain injuries in cerebrospinal fluid using blood-validated ck and ck-mb immunoassays. Biomolecules.

[30] Olczak M (2018). Brain-originated peptides as possible biochemical markers of traumatic brain injury in cerebrospinal fluid post-mortem examination. Folia Neuropathol..

[31] Akçan R, Taştekin B, Yildirim M, Aydogan HC, Sağlam N (2020). Omics era in forensic medicine: Towards a new age. Turk. J. Med. Sci..

[32] Szeremeta M, Pietrowska K, Niemcunowicz-Janica A, Kretowski A, Ciborowski M (2021). Applications of metabolomics in forensic toxicology and forensic medicine. Int. J. Mol. Sci..

[33] Du TS (2020). Research progress of metabolomics in forensic pathology. Fa Yi Xue Za Zhi.

[34] Castillo-Peinado LS, Luque de Castro MD (2016). Present and foreseeable future of metabolomics in forensic analysis. Anal. Chim. Acta.

[35] Scow RO, Blanchette-Mackie EJ, Smith LC (1980). Transport of lipid across capillary endothelium. Fed. Proc..

[36] van der Vusse GJ, van Bilsen M, Glatz JF (2000). Cardiac fatty acid uptake and transport in health and disease. Cardiovasc. Res..

[37] Kampf JP, Parmley D, Kleinfeld AM (2007). Free fatty acid transport across adipocytes is mediated by an unknown membrane protein pump. Am. J. Physiol. Endocrinol. Metab..

[38] Abumrad NA, Perkins RC, Park JH, Park CR (1981). Mechanism of long chain fatty acid permeation in the isolated adipocyte. J. Biol. Chem..

[39] Luiken JJ (1999). Cellular fatty acid transport in heart and skeletal muscle as facilitated by proteins. Lipids.

[40] Stremmel W (1988). Fatty acid uptake by isolated rat heart myocytes represents a carrier-mediated transport process. J. Clin. Invest..

[41] Heather LC (2006). Fatty acid transporter levels and palmitate oxidation rate correlate with ejection fraction in the infarcted rat heart. Cardiovasc. Res..

[42] Rosenblatt-Velin N, Montessuit C, Papageorgiou I, Terrand J, Lerch R (2001). Postinfarction heart failure in rats is associated with upregulation of glut-1 and downregulation of genes of fatty acid metabolism. Cardiovasc. Res..

[43] Allard MF (2004). Energy substrate metabolism in cardiac hypertrophy. Curr. Hypertens. Rep..

[44] Stanley WC, Recchia FA, Lopaschuk GD (2005). Myocardial substrate metabolism in the normal and failing heart. Physiol. Rev..

[45] Djoussé L (2013). Plasma free fatty acids and risk of heart failure: The cardiovascular health study. Circ. Heart Fail..

[46] Taylor M (2001). An evaluation of myocardial fatty acid and glucose uptake using pet with [18f]fluoro-6-thia-heptadecanoic acid and [18f]fdg in patients with congestive heart failure. J. Nucl. Med..

[47] Voros G (2018). Increased cardiac uptake of ketone bodies and free fatty acids in human heart failure and hypertrophic left ventricular remodeling. Circ. Heart Fail..

[48] Cheng ML (2015). Metabolic disturbances identified in plasma are associated with outcomes in patients with heart failure: Diagnostic and prognostic value of metabolomics. J. Am. Coll. Cardiol..

[49] Tenori L (2013). Metabolomic fingerprint of heart failure in humans: A nuclear magnetic resonance spectroscopy analysis. Int. J. Cardiol..

[50] Turer AT (2009). Metabolomic profiling reveals distinct patterns of myocardial substrate use in humans with coronary artery disease or left ventricular dysfunction during surgical ischemia/reperfusion. Circulation.

[51] Desmoulin F (2013). Metabonomics analysis of plasma reveals the lactate to cholesterol ratio as an independent prognostic factor of short-term mortality in acute heart failure. PLoS One.

[52] Phillis JW, Horrocks LA, Farooqui AA (2006). Cyclooxygenases, lipoxygenases, and epoxygenases in cns: Their role and involvement in neurological disorders. Brain Res. Rev..

[53] Svennerholm L (1968). Distribution and fatty acid composition of phosphoglycerides in normal human brain. J. Lipid Res..

[54] Svennerholm L, Vanier MT (1972). The distribution of lipids in the human nervous system. Ii. Lipid composition of human fetal and infant brain. Brain Res..

[55] Han X (2007). Neurolipidomics: Challenges and developments. Front. Biosci..

[56] Zhang J (2012). Traumatic brain injury-associated coagulopathy. J. Neurotrauma.

[57] Pasvogel AE, Miketova P, Moore IM (2010). Differences in csf phospholipid concentration by traumatic brain injury outcome. Biol. Res. Nurs..

[58] Sarkar C (2020). Pla2g4a/cpla2-mediated lysosomal membrane damage leads to inhibition of autophagy and neurodegeneration after brain trauma. Autophagy.

[59] Yan EB (2015). Activation of the kynurenine pathway and increased production of the excitotoxin quinolinic acid following traumatic brain injury in humans. J. Neuroinflammation.

[60] Meier TB, Savitz J (2022). The kynurenine pathway in traumatic brain injury: Implications for psychiatric outcomes. Biol. Psychiatry.

[61] Huang J (2020). Spermidine exhibits protective effects against traumatic brain injury. Cell. Mol. Neurobiol..

[62] Madeo F, Eisenberg T, Pietrocola F, Kroemer G (2018). Spermidine in health and disease. Science.

[63] Minois N (2014). Molecular basis of the ‘anti-aging’ effect of spermidine and other natural polyamines - A mini-review. Gerontology.

[64] Bayir H (2002). Assessment of antioxidant reserves and oxidative stress in cerebrospinal fluid after severe traumatic brain injury in infants and children. Pediatr. Res..

[65] Meister A (1992). On the antioxidant effects of ascorbic acid and glutathione. Biochem. Pharmacol..

[66] Drake J, Kanski J, Varadarajan S, Tsoras M, Butterfield DA (2002). Elevation of brain glutathione by gamma-glutamylcysteine ethyl ester protects against peroxynitrite-induced oxidative stress. J. Neurosci. Res..

[67] Tyurin VA (2000). Oxidative stress following traumatic brain injury in rats: Quantitation of biomarkers and detection of free radical intermediates. J. Neurochem..

[68] Hannun YA, Obeid LM (2002). The ceramide-centric universe of lipid-mediated cell regulation: Stress encounters of the lipid kind. J. Biol. Chem..

[69] Obeid LM, Linardic CM, Karolak LA, Hannun YA (1993). Programmed cell death induced by ceramide. Science.

[70] García-Arribas AB, Alonso A, Goñi FM (2016). Cholesterol interactions with ceramide and sphingomyelin. Chem. Phys. Lipids.

[71] Palafox-Sánchez V, Ying Z, Royes LFF, Gomez-Pinilla F (2021). The interaction between brain and liver regulates lipid metabolism in the tbi pathology. Biochim. Biophys. Acta Mol. Basis Dis..

[72] Yue JT (2015). A fatty acid-dependent hypothalamic-dvc neurocircuitry that regulates hepatic secretion of triglyceride-rich lipoproteins. Nat. Commun..

[73] Taher J, Farr S, Adeli K (2017). Central nervous system regulation of hepatic lipid and lipoprotein metabolism. Curr. Opin. Lipidol..

[74] Nizamutdinov D (2017). Hepatic alterations are accompanied by changes to bile acid transporter-expressing neurons in the hypothalamus after traumatic brain injury. Sci. Rep..

[75] Liu P (2020). The mechanisms of lysophosphatidylcholine in the development of diseases. Life Sci..

[76] Mousavi Majd A (2018). Inhibition of gaba a receptor improved spatial memory impairment in the local model of demyelination in rat hippocampus. Behav. Brain Res..

[77] Boulard G (2001). Plasma osmolarity and cerebral volume. Ann. Fr. Anesth. Reanim..

[78] Zhong YH (2021). Serum levels of hdl cholesterol are associated with diffuse axonal injury in patients with traumatic brain injury. Neurocrit. Care.

[79] Merkley ED, Wunschel DS, Wahl KL, Jarman KH (2019). Applications and challenges of forensic proteomics. Forensic Sci. Int..

[80] ThermoFisherScientific. *Q exactive™ Plus Hybrid Quadrupol-Orbitrap™ Massenspektrometer*, https://www.thermofisher.com/order/catalog/product/IQLAAEGAAPFALGMBDK?SID=srch-srp-IQLAAEGAAPFALGMBDK (2023).

[81] Bligh EG, Dyer WJ (1959). A rapid method of total lipid extraction and purification. Can. J. Biochem. Physiol..

