# Supplementary material for: Neuroforensomics: metabolites as valuable biomarkers in cerebrospinal fluid of lethal traumatic brain injuries
Source: Sci Rep. 2024 Jun 13;14:13651. doi: 10.1038/s41598-024-64312-0 (PMC11176198; doi:10.1038/s41598-024-64312-0)
Supplement: Supplementary file 1 — Supplementary Information. [file 41598_2024_64312_MOESM1_ESM.pdf]

Supplementary material to:

Neuroforensomics: Metabolites as valuable biomarkers in cerebrospinal fluid of lethal traumatic brain injuries

Simone Bohnert[1], Christoph Reinert[1], Stefanie Trella[1], Andrea Cattaneo[2]
Ulrich Preiß[1], Michael Bohnert[1], Johann Zwirner [3,4], Andreas Büttner[5], Werner Schmitz[6], Benjamin Ondruschka[3*]

[1]Institute of Forensic Medicine, University of Würzburg, Würzburg, Germany

[2]Department of Neurosurgery, University Hospital Würzburg, Würzburg, Germany

[3]Institute of Legal Medicine, University Medical Center Hamburg-Eppendorf, Hamburg, Germany

[4]Department of Oral Sciences, University of Otago, Dunedin, New Zealand

[5]Institute of Forensic Medicine, Rostock University Medical Center, Rostock, Germany

[6]Institute of Biochemistry and Molecular Biology, Biozentrum, University of Würzburg, Würzburg, Germany

*Correspondence should be addressed to B.O. (e-mail: b.ondruschka@uke.de) and requests for materials should be addressed to S.B. (e-mail: simone.bohnert@uni-wuerzburg.de).

S.B. and C.R. contributed as first authors equally.

Case number	Sex	Age	Post mortem interval	Cause of Death	Cardiopulmonary resuscitation	Brain weight
	[m/f]	[years]	[hours]	[TBI/CVF]	[Yes/No]	[grams]
**1**	f	56	93	CVF	Yes	1445
**2**	m	58	139	CVF	No	1565
**3**	m	59	219	CVF	Yes	1525
**4**	m	57	49	CVF	Yes	1650
**5**	m	38	179	CVF	Yes	1520
**6**	m	48	111	CVF	No	1670
**7**	m	49	177	CVF	Yes	1455
**8**	m	59	151	CVF	Yes	1640
**9**	f	82	155	CVF	Yes	1335
**10**	m	88	72	CVF	No	1250
**11**	m	84	112	CVF	Yes	1500
**12**	m	64	48	CVF	Yes	1540
**13**	f	85	69	CVF	Yes	1190
**14**	m	79	121	CVF	Yes	1420
**15**	f	86	71	CVF	Yes	1300
**16**	f	78	82	CVF	No	1000
**17**	f	78	44	CVF	No	1130
**18**	m	84	118	CVF	No	1535
**19**	m	68	35	CVF	No	1250
**20**	m	83	69	CVF	Yes	1380
**21**	f	93	198	CVF	No	1120
**22**	f	84	148	CVF	No	1210
**23**	f	66	63	CVF	Yes	1440
**24**	m	91	45	CVF	No	1200
**25**	m	81	109	CVF	No	1470
**26**	f	76	80	CVF	Yes	1200
**27**	m	35	67	CVF	Yes	1170
**28**	m	57	309	CVF	Yes	1620
**29**	m	54	127	CVF	Yes	1395
**30**	m	33	59	CVF	Yes	1640
**31**	m	78	161	TBI	Yes	1500
**32**	f	77	152	TBI	No	1060
**33**	m	83	88	TBI	Yes	1320
**34**	f	87	129	TBI	Yes	1220
**35**	m	36	153	TBI	No	1320
**36**	m	54	120	TBI	Yes	1410
**37**	m	92	44	TBI	No	1130
**38**	m	82	37	TBI	No	1310
**39**	m	66	185	TBI	No	1380
**40**	m	88	141	TBI	No	1655
**41**	m	83	93	TBI	No	1305
**42**	m	55	32	TBI	No	1235
**43**	f	80	123	TBI	No	1235
**44**	m	42	109	TBI	Yes	1410
**45**	f	63	130	TBI	Yes	1275
**46**	m	88	93	TBI	No	1180

**47**	m	66	184	TBI	No	1560
**48**	f	65	39	TBI	No	1240
**49**	m	22	141	TBI	No	1390
**50**	m	82	93	TBI	No	1470
**51**	m	75	164	TBI	No	1480
**52**	m	55	117	TBI	Yes	1440
**53**	f	36	93	TBI	Yes	1360
**54**	m	19	170	TBI	Yes	1540
**55**	f	57	199	TBI	Yes	1140
**56**	f	87	184	TBI	No	1190
**57**	f	80	66	TBI	No	1080
**58**	m	87	188	TBI	Yes	1380
**59**	m	83	182	TBI	No	1320
**60**	m	31	157	TBI	Yes	1595

Supplementary table 1*: Case characteristics of the study. Traumatic brain injury (TBI); cardiovascular fatalities (CVF); f (female), m (male).*

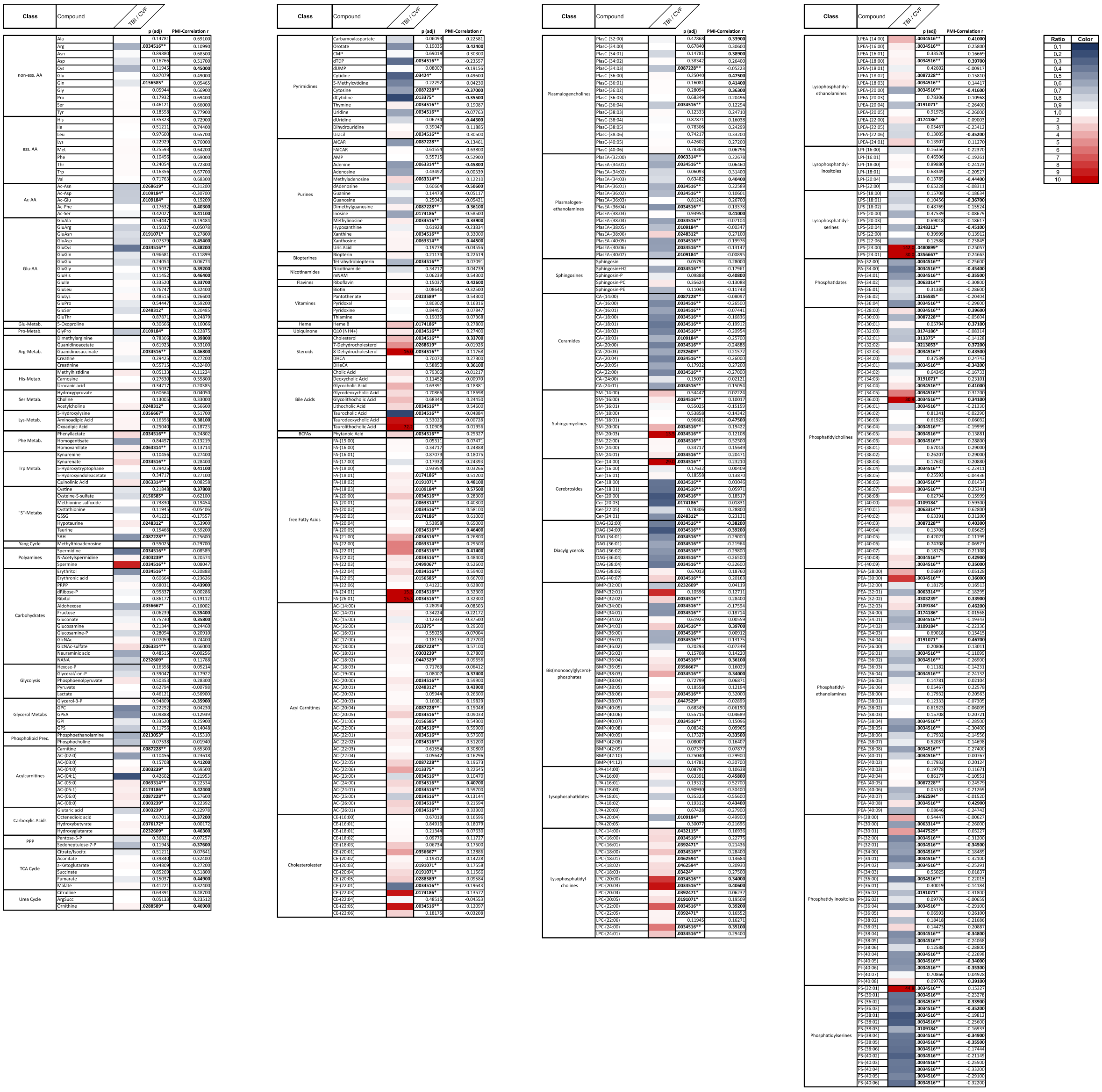

***Supplementary figure 1**: Heatmap of all tested metabolites between traumatic brain injury (TBI) and cardiovascular failure (CVF) as cause of death. Data was checked for differences between TBI and CVF as well as correlation of metabolites level to post mortem interval (PMI).*
